# Stroke and Bleeding Risks in Atrial Fibrillation Are Not Static but Dynamic: A Dynamic Assessment of CHA_2_DS_2_‐VASc and HAS‐BLED Scores in the Iranian Registry of Atrial Fibrillation (IRAF)

**DOI:** 10.1111/jce.70190

**Published:** 2025-11-20

**Authors:** Amir Askarinejad, Tommaso Bucci, Enrico Tartaglia, Michele Rossi, Gregory Y. H. Lip, Majid Haghjoo

**Affiliations:** ^1^ Liverpool Centre for Cardiovascular Sciences at University of Liverpool, Liverpool John Moores University and Liverpool Heart & Chest Hospital Liverpool UK; ^2^ Internal, Vascular and Emergency Medicine – Stroke Unit University of Perugia Perugia Italy; ^3^ Department of Clinical Medicine Aalborg University Aalborg Denmark; ^4^ Medical University of Bialystok Bialystok Poland; ^5^ Department of Electrophysiology Rajaie Cardiovascular Medical and Research Center, Iran university of medical sciences Tehran Iran; ^6^ Cardiac Electrophysiology Research Center, Rajaie Cardiovascular Medical and Research Center Iran University of Medical Sciences Tehran Iran

**Keywords:** atrial fibrillation, bleeding risk, haemorragic risk, stroke, stroke risk

## Abstract

**Background and Objectives:**

Proper stroke and bleeding risk assessment is an essential part of clinical decision‐making in patients with atrial fibrillation (AF). This study aims to determine whether the dynamic assessment of CHA_2_DS_2_‐VASc and HAS‐BLED scores over time enhances stroke and bleeding risk prediction.

**Methods:**

In this prospective longitudinal study—based on data from the Iranian Atrial Fibrillation Registry (IRAF)—patients with available CHA₂DS₂‐VASc and HAS‐BLED scores at baseline, first follow‐up (after 6 months), and second follow‐up (after 12 months) were included. Univariable and multivariable logistic regression analyses were performed to evaluate the association of CHA₂DS₂‐VASc and HAS‐BLED scores (i.e., scores at baseline, first follow‐up, and their difference defined as delta) with stroke and bleeding risk, respectively.

**Results:**

A total of 529 patients (mean age 61.2 ± 14.3 years; 59.7% male) were included. CHA₂DS₂‐VASc and HAS‐BLED scores increased significantly after 6 and 12 months from recruitment. *delta* CHA₂DS₂‐VASc score (AUC 0.69; 95% CI: 0.54–0.84) and *First follow‐up* CHA₂DS₂‐VASc score (AUC 0.70; 95% CI: 0.51–0.88) achieved higher AUCs compared to *baseline* CHA₂DS₂‐VASc score (AUC 0.63; 95% CI: 0.44–0.82). *delta* HAS‐BLED score had the highest AUC (AUC 0.73; 95% CI: 0.55–0.91) compared to *baseline* (AUC 0.58; 95% CI: 0.43–0.74) and *first follow‐up* HAS‐BLED (AUC 0.60; 95% CI: 0.44–0.80) scores.

**Conclusions:**

Regular reassessment of CHA₂DS₂‐VASc and HAS‐BLED scores is essential in AF patients, as stroke and bleeding risks change over time, emphasizing the need for dynamic risk stratification.

**Abbreviations**
AF: atrial fibrillation
AUC: area under the curve
CI: confidene interval
CKD: chronic kidney disease
IRAF: Iranian Atrial Fibrillation Registry
LVEF: left ventricular ejection fraction
OACs: oral anticoagulants
OR: odds ratio
ROC: receiver operating characteristic

## 1 Introduction

Atrial fibrillation (AF) is the most prevalent cardiac arrhythmia, significantly contributing to morbidity and mortality due to complications such as stroke, thromboembolism, heart failure, and reduced quality of life [1]. AF has emerged as a global public health challenge, affecting around 52.6 million people globally in 2021, with a substantial increase in prevalence, mortality, and disability‐adjusted life years since 1990 [2]. The use of oral anticoagulants (OACs) in AF patients has been associated with a reduced risk of both stroke and all‐cause mortality, but achieving an optimal balance between stroke prevention and the risk of bleeding remains a significant clinical challenge [3].

Current clinical risk scores rely on a “static” risk assessment approach, evaluating baseline risk factors to predict events occurring years later. However, stroke risk in AF is dynamic, evolving over time due to aging and the development of new comorbidities [4]. Evidence suggests that the majority of patients with AF will develop at least one new risk factor before experiencing a thromboembolic event [5]. Dynamic changes in patient health status can lead to increases in CHA₂DS₂‐VASc and HAS‐BLED scores over time, altering the absolute risk of stroke and bleeding. Consequently, the initially estimated risk may worsen due to aging and the development of new comorbidities [6].

There is a significant gap in research on AF as well as the dynamic progression of CHA₂DS₂‐VASc and HAS‐BLED scores in AF patients in the Middle East. Understanding these changes over time is crucial for improving risk prediction and patient management in this region.

This study aimed to evaluate the predictive ability of baseline and longitudinal changes in CHA₂DS₂‐VASc and HAS‐BLED scores for stroke and bleeding events in patients with AF, respectively.

## 2 Methods

This prospective longitudinal study was based on data from the Iranian Atrial Fibrillation Registry (IRAF), a nationwide cohort established to monitor and analyze AF patients over time [7, 8]. The IRAF registry includes patients diagnosed with AF, with structured follow‐ups conducted at 6 months and 1 year to assess disease progression, treatment outcomes, and associated complications. The methodology for patient recruitment, data collection, and registry details has been previously documented in published studies [7, 8]. For the present analysis, we included AF patients enrolled in the IRAF registry who had complete data on CHA₂DS₂‐VASc and HAS‐BLED scores at baseline and at the 6‐month follow‐up. Patients were excluded if they lacked follow‐up data at the 1‐year mark, as continuous assessment was necessary for evaluating dynamic risk changes. Additionally, individuals with incomplete baseline and follow‐up data or missing essential clinical variables were not considered in the final analysis to ensure data integrity and the reliability of predictive modeling.

### 2.1 Variables and Follow‐ups

Demographic data, symptoms, comorbidities, and clinical risk scores including CHA₂DS₂‐VASc and HAS‐BLED scores were collected though patient's medical records. Bleeding events were defined as the occurrence of any major bleeding (including gastrointestinal and intracranial bleeding), clinically relevant nonmajor bleeding (such as epistaxis), and minor bleeding (including bruising or superficial hematoma).

CHA₂DS₂‐VASc score was assessed at baseline (*baseline* CHA₂DS₂‐VASc score), 6‐months (*first follow‐up* CHA₂DS₂‐VASc score), and after 12‐months (*second follow‐up* CHA₂DS₂‐VASc score). *Delta* CHA₂DS₂‐VASc score was calculated from subtracting *baseline* CHA₂DS₂‐VASc score from *first follow‐up* CHA₂DS₂‐VASc score.

The HAS‐BLED score was assessed at baseline (*baseline* HAS‐BLED score), 6‐months (*first follow‐up* HAS‐BLED score), and after 12‐months (*second follow‐up* HAS‐BLED score). *Delta* HAS‐BLED score was calculated from subtracting *baseline* HAS‐BLED score from *first follow‐up* HAS‐BLED score. All patients had first follow‐up after 6 months and second follow‐up after 12 months from enrollment.

### 2.2 Outcomes

The primary outcome was the dynamic changes of CHA₂DS₂‐VASc and HAS‐BLED scores during baseline, first follow‐up and second follow‐up. The secondary outcomes were as follows: (i) The association of *baseline*, *first follow‐up*, and *delta* CHA₂DS₂‐VASc scores with stroke risk; (ii) The association of *baseline*, *first follow‐up*, and *delta* HAS‐BLED scores with bleeding risk; (iii) The predictive performance of *first follow‐up*, and *delta* CHA₂DS₂‐VASc scores with *baseline* CHA₂DS₂‐VASc score to predict stroke risk; (iv) The predictive performance of *first follow‐up*, and *delta* HAS‐BLED scores with *baseline* HAS‐BLED score to predict bleeding risk; (v) stroke risk in patients who recently transitioned to a higher CHA₂DS₂‐VASc score compared with those who were stably high risk since baseline; (vi) bleeding risk according to change in HAS‐BLED score category (stable, upward transition, and downward transition).

Bleeding and stroke risk were defined as bleeding and stroke events that have been occurred from first follow‐up to second follow‐up.

### 2.3 Statistical Analysis

#### 2.3.1 Descriptive Analysis

Numerical data were presented as mean ± standard deviation (SD) and categorical data were reported as frequency and percentage (*N*, %). Paired *t*‐test was used to assess the significance difference of means of CHA₂DS₂‐VASc and HAS‐BLED scores from baseline to 6‐month and 1‐year follow‐ups. To visually represent score transitions over time, alluvial diagrams were generated to illustrate changes in CHA₂DS₂‐VASc and HAS‐BLED scores from baseline to 6‐month and 1‐year follow‐ups using *ggplot2* and *ggalluvial* packages.

#### 2.3.2 Survival Analaysis

Univariable and multivariate logistic regression models were utilized to examine the association between CHA₂DS₂‐VASc and HAS‐BLED scores and the risk of stroke and bleeding, respectively. Both scores were entered into the logistic regression models as continuous variables, and the reported odds ratios reflect the effect of each incremental point increase in these scores. Multivariate logistic regression models for predicting stroke were adjusted for potential confounders including AF type (i.e., paroxysmal, persistent, or permanent), left atrial diameter, left ventricular ejection fraction (LVEF), chronic kidney disease (CKD), and OAC use. Multivariate logistic regression models for predicting bleeding events were adjusted for potential confounders to identify independent predictors of adverse outcomes including age, gender, LVEF, and OAC use. Three multivariable logistic regression models were constructed for both stroke and bleeding outcomes: (i) Model I included the baseline CHA₂DS₂‐VASc (or HAS‐BLED) score together with all covariates (AF type, left atrial diameter, left ventricular ejection fraction, chronic kidney disease, oral anticoagulant use, age, and sex); (ii) model II incorporated the first follow‐up score (6 months) with the same covariates; (iii) model III included the absolute change in score (delta CHA₂DS₂‐VASc or delta HAS‐BLED) between baseline and 12 months, adjusted for the same covariates.

Odds ratios (ORs) with corresponding 95% confidence intervals (CIs) were derived from each model to quantify the association between incremental score changes and clinical outcomes.

Receiver operating characteristic (ROC) curve analysis was performed to compare the predictive accuracy of baseline, first follow‐up, and delta scores. The area under the curve (AUC) was used to quantify the discriminative ability of these risk scores in predicting stroke and bleeding events. Delong's test was used to assess the statistically significant difference among AUCs of *baseline*, *first follow‐up*, and *delta* CHA₂DS₂‐VASc and HAS‐BLED scores [9]. A two‐sided *p*‐value of less than 0.05 was considered statistically significant.

#### 2.3.3 Multistate Model Analysis

To examine whether patients who recently transitioned to a higher CHA₂DS₂‐VASc score were at greater risk of stroke compared with those who were stably high risk, we performed a multistate model analysis. Patients were classified into two mutually exclusive categories: (i) *Stable High Stroke Risk since Baseline* (already high risk (CHA₂DS₂‐VASc score > 2) at baseline and at 6 months) and (ii) *New high by 6 months* (low (CHA₂DS₂‐VASc score = 0) or intermediate (CHA₂DS₂‐VASc score = 1) at baseline, high (CHA₂DS₂‐VASc score > 2) at 6 months). Multivariable logistic regression was used to estimate ORs and 95% CIs for each exposure group, with “*Stable high*” as the reference category. Model was adjusted for AF type (paroxysmal, persistent, permanent), CKD, OAC at enrollment, and LVEF.

To examine the association between change in HAS‐BLED score categories and 1‐year risk of bleeding, we performed a multistate model analysis. Patients were classified into three mutually exclusive categories: (i) *Stable bleeding Risk* (no change in bleeding risk category from baseline to first follow‐up); (ii) *upward transition in bleeding risk* (from low (HAS‐BLED score = 0) to intermediate (HAS‐BLED score = 1–2) or from intermediate to high risk (HAS‐BLED score > 3)) at first follow‐up); (iii) *downward transition in bleeding risk* (transition from high to intermediate or from intermediate to low risk). Multivariable logistic regression was used to estimate ORs and 95% CIs for each exposure group, with “*Stable bleeding Risk*” as the reference category. Models were adjusted for AF type (paroxysmal, persistent, permanent), OAC at enrollment, LVEF, age, and gender. Because events were infrequent in multistate analysis, Firth's penalised likelihood logistic regression was applied [10].

All statistical analyses were conducted using R software version 4.4.1 (R Core Team, Vienna, Austria).

## 3 Results

**Figure 1:**
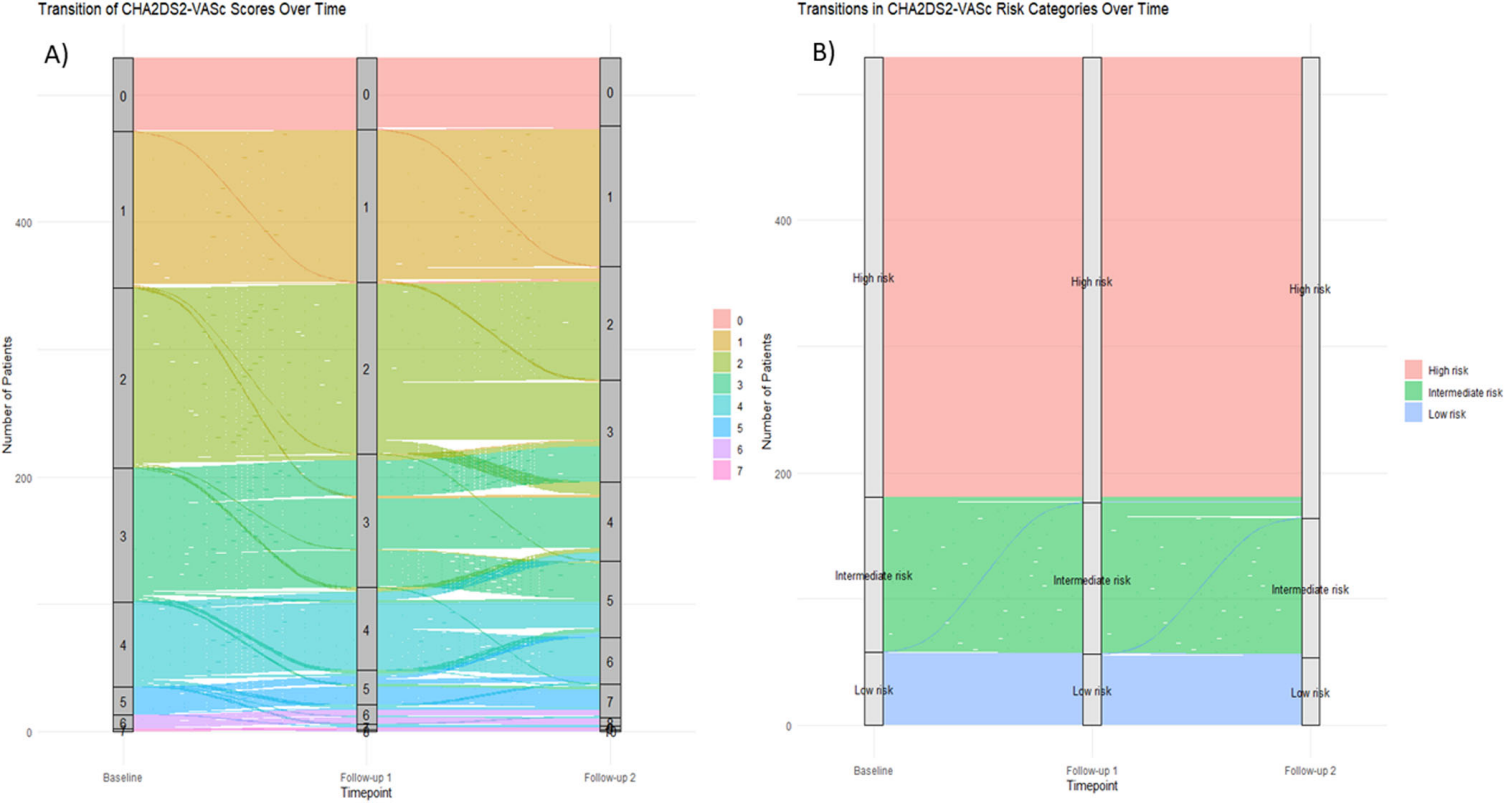
CHA₂DS₂‐VASc score transition patterns at baseline, first, and second follow‐up. (A) Demonstrates the changes CHA₂DS₂‐VASc Score during baseline, first follow‐up and second follow‐up. (B) Demonstrates the changes of CHA₂DS₂‐VASc Score categories during baseline, first follow‐up and second follow‐up.

A total of 529 patients (mean age 61.2 ± 14.3 years; 59.7% male) were included in the study (Supporting Information S1: Figure 1). Hypertension, hyperlipidaemia, and diabetes were observed in 272 (51.4%), 129 (24.4%), and 103 (19.5%) of patients. The mean value of CHA_2_DS_2_‐VASc and HAS‐BLED scores were 2.2 and 1.1, respectively. Baseline characteristics of the study population are summarised Table 1.

**Table 1:** Baseline clinical and demographic features of the study population.

Variable		Baseline
Age		61.2 ± 14.3
Male		316 (59.7)
AF type	Paroxysmal	368 (69.5)
	Persistent	120 (22.7)
	permanent	41 (7.8)
Symptoms	Palpitations	354 (66.9)
	Dyspnea	140 (26.5)
	Chest pain	4 (0.8)
	Dizziness	40 (7.6)
	Syncope	13 (2.5)
	Fatigue	20 (3.8)
	Anxiety	9 (1.7)
Comorbidities	Hypertension	272 (51.4)
	Hyperlipidemia	129 (24.4)
	Diabetes	103 (19.5)
	Smoking	28 (5.3)
	Alcohol	6 (1.1)
	Opium	2 (0.4)
	Heart Failure	99 (18.7)
	Chronic Kidney disease	33 (6.2)
	Coronary artery disease	96 (18.1)
	Myocardial infarction	18 (3.4)
Echocardiographic findings	LA diameter (mm)	5.1 ± 2.6
	LVEF (%)	43.4 ± 13.1
OAC use	VKAs	155 (29.3)
	DOACs	219 (41.4)
Clinical risk scores	CHA_2_DS_2_‐VASc score	2.2 ± 1.5
	HAS‐BLED score	1.2 ± 0.9

Abbreviations: LA, left atrium; LVEF, left ventricular ejection fraction.

### 3.1 Temporal Changes in CHA₂DS₂‐VASc and HAS‐BLED Scores

From baseline to first follow‐up 16 (3.0%) and 4 (0.8%) patients experienced stroke and bleeding events, respectively.

The CHA₂DS₂‐VASc score significantly increased from baseline to first follow‐up (2.2 ± 1.5 to 2.3 ± 1.6) and from first follow‐up to second follow‐up (2.3 ± 1.6 to 3.9 ± 2.1). From baseline to 6 months, only 1.7% progressed to Intermediate stroke risk (CHA₂DS₂‐VASc score = 1) and 1.7% to High risk (CHA₂DS₂‐VASc score ≥ 2), while the majority (96.6%) remained Low (CHA₂DS₂‐VASc score = 0). Between 6 and 12 months, 3.6% moved to Intermediate and 1.8% to High risk, with 94.6% remaining Low.

**Figure 2:**
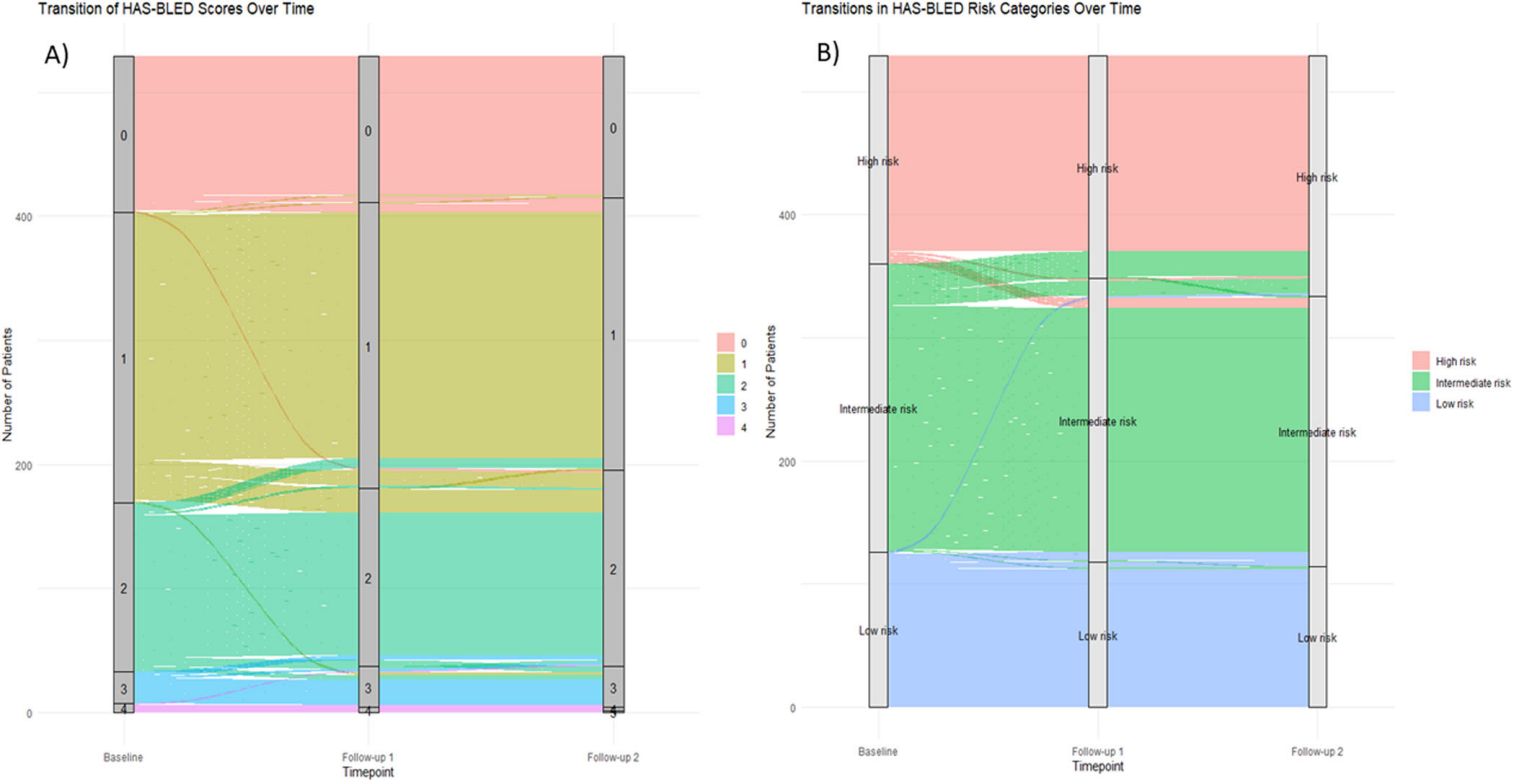
HAS‐BLED score transition patterns at baseline, first, and second follow‐up. (A) Demonstrates the changes HAS‐BLED Score during baseline, first follow‐up and second follow‐up. (B) Demonstrates the changes of HAS‐BLED Score categories during baseline, first follow‐up and second follow‐up.

HAS‐BLED score significantly increased from baseline (1.1 ± 0.9) to first follow‐up (1.2 ± 0.9) (Table 2). Figures 1 and 2 illustrates temporal evolution of CHA₂DS₂‐VASc and HAS‐BLED scores across sequential follow‐up periods, respectively. From baseline to 6 months, 1.5% of patients progressed to a higher bleeding risk category, while 0.9% improved to a lower risk category, with the majority (97.6%) remaining in the same HAS‐BLED category. Between 6 and 12 months, 0.8% shifted to a higher risk category, 1.0% to a lower risk category, and 98.2% remained stable overall. From baseline to 6 months, the HAS‐BLED score decreased in 3.4% of patients, increased in 7.0%, and remained stable in 89.6%. Between 6 and 12 months, 1.5% showed a decrease, 5.1% an increase, and 93.4% remained unchanged.

**Table 2:** HAS‐BLED and CHA₂DS₂‐VASc scores at baseline, first follow‐up, and second follow‐ups.

Score		Baseline (*N* = 529)	First follow‐up (*N* = 529)	Second follow‐up (*N* = 529)	*p*‐value
CHA_2_DS_2_‐VASc score	0 (low risk)	58	56	53	0 < 0.001* 0 < 0.001**
	1 (intermediate risk)	123	120	111	
	≥ 2 (high risk)	348	355	365	
	Mean value	2.2 ± 1.5	2.3 ± 1.6	3.0 ± 2.1	0 < 0.001* 0 < 0.001**
HAS‐BLED score	0 (low risk)	126	118	114	0 < 0.001* 0 < 0.001**
	1–2 (intermediate risk)	370	374	378	
	≥ 3 (high risk)	33	37	37	
	Mean value	1.1 ± 0.9	1.2 ± 0.9	1.2 ± 0.9	0 < 0.001*, 0.003**

* Baseline vs. first follow‐up;

** First‐follow‐up vs. second follow‐up.

### 3.2 Survival Analysis

From first follow‐up to second follow‐up, 12 (2.3%) and 6 (1.1%) patients experienced stroke and bleeding, respectively.

*First follow‐up* CHA₂DS₂‐VASc score was significantly associated with an increased risk of stroke in both univariate (OR 1.64, 95% CI 1.19–2.26) and multivariable (OR 1.72, 95% CI 1.21–2.43) regression analysis (Table 3). *Delta* CHA₂DS₂‐VASc score was a significant predictor of stroke in both univariate (OR 4.28, 95% CI 2.36–7.78) and multivariable (OR 4.24, 95% CI 2.26–7.94) regression analysis (Table 3).

**Table 3:** Univariate and multivariable logistic regression analysis for stroke and bleeding risk.

			Univariate analysis for stroke, OR (95% CI)	Multivariable analysis for stroke, OR (95% CI)		
				Model I	Model II	Model III
Baseline CHA₂DS₂‐VASc score			1.31 (0.91–1.88)	1.35 (0.90–2.02)	NA	NA
First follow‐up CHA₂DS₂‐VASc score			1.64 (1.19–2.26)	NA	1.72 (1.21–2.43)	NA
Delta CHA₂DS₂‐VASc score			4.28 (2.36–7.78)	NA	NA	4.24 (2.26–7.94)
AF type	Paroxysmal		Ref	Ref	Ref	Ref
	Persistent		0.68 (0.14–3.17)	0.52 (0.10–2.54)	0.44 (0.08–2.27)	0.47 (0.08–2.73)
	Permanent		1.00 (0.12–8.08)	0.76 (0.09–6.55)	0.70 (0.08–6.01)	1.25 (0.14–10.94)
LA diameter			0.98 (0.84–1.13)	0.96 (0.83–1.12)	0.96 (0.82–1.13)	0.95 (0.82–1.11)
LVEF			0.98 (0.94–1.02)	0.98 (0.94–1.02)	0.99 (0.95–1.04)	0.99 (0.94–1.03)
CKD			1.38 (0.17–11.01)	0.90 (0.10–8.07)	0.82 (0.09–7.40)	1.78 (0.21–15.36)
OAC use			0.40 (0.12–1.34)	0.32 (0.09–1.10)	0.28 (0.08–1.00)	0.39 (0.11–1.41)

			Univariate analysis for bleeding, OR (95% CI)	Multivariable analysis for bleeding, OR (95% CI)		
				Model I	Model II	Model III
Baseline HAS‐BLED score			0.67 (0.28–1.60)	0.22 (0.03–1.56)	NA	NA
First follow‐up HAS‐BLED score			1.45 (0.69–3.08)	NA	2.20 (0.77–6.27)	NA
Delta HAS‐BLED score			11.00 (3.43–35.32)	NA	NA	11.11 (3.28–37.60)
Age			1.07 (0.98–1.16)	1.07 (0.98–1.16)	0.99 (0.93–1.06)	1.01 (0.95–1.07)
Gender		Male	Ref	Ref	Ref	Ref
		Female	1.59 (0.39–6.51)	1.46 (0.35–6.05)	1.46 (0.35–6.05)	1.31 (0.30–5.80)
LVEF			1.04 (0.96–1.12)	1.04 (0.96–1.12)	1.05 (0.97–1.13)	1.05 (0.97–1.14)
OAC use			0.81 (0.20–3.27)	3.82 (0.36–40.94)	0.39 (0.07–2.32)	0.90 (0.20–3.95)

*Note:* Model I included the baseline CHA₂DS₂‐VASc (or HAS‐BLED) score together with all covariates (AF type, left atrial diameter, left ventricular ejection fraction, chronic kidney disease, oral anticoagulant use, age, and sex); model II incorporated the first follow‐up score (6 months) with the same covariates; model III included the absolute change in score (delta CHA₂DS₂‐VASc or delta HAS‐BLED) between baseline and 12 months, adjusted for the same covariates.

Abbreviations: AF, atrial fibrillation; CI, confidence interval; CKD, chronic kidney disease; LA, left atrium; LVEF, left ventricular ejection fraction; OAC, oral anticoagulant; OR, odds ratio.

*First follow‐up* HAS‐BLED score was associated with an increased risk of bleeding in both univariate (OR 1.45, 95% CI 0.69–3.08) and multivariable (OR 2.20, 95% CI 0.77–6.27) regression analysis, although this association did not reach statistical significance (Table 3). *Delta* HAS‐BLED score was a significant predictor of bleeding events in both univariate (OR 11.00, 95% CI 3.43–35.32) and multivariable (OR 11.11, 95% CI 3.28–37.60) regression analysis (Table 3).

### 3.3 Comparing the Predictive Performance of Baseline, First Follow up, and Delta CHA₂DS₂‐VASc Scores

**Figure 3:**
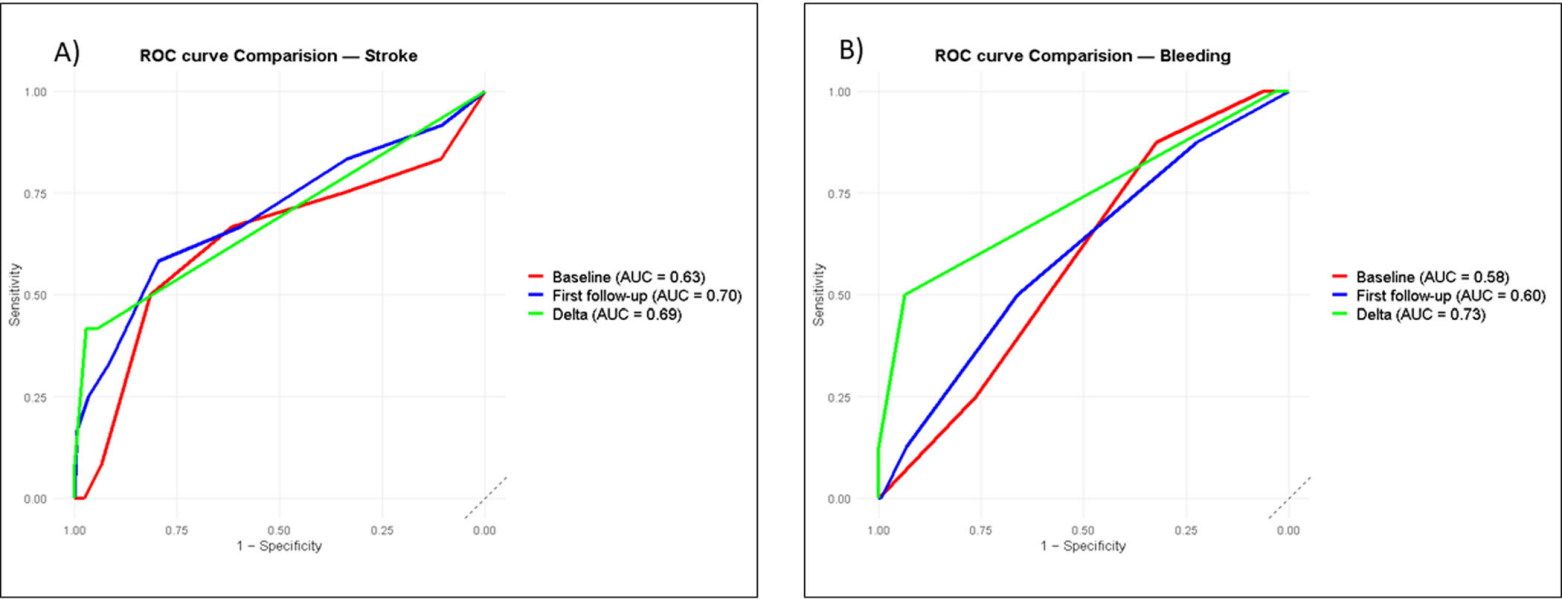
Receiver Operating Characteristic (ROC) Curve Comparison for baseline CHA₂DS₂‐VASc score, first follow‐up CHA₂DS₂‐VASc score, and delta CHA₂DS₂‐VASc score in Predicting Stroke Risk (A) and for baseline HAS‐BLED Score, first follow‐up HAS‐BLED Score, and delta HAS‐BLED Score in Predicting bleeding events (B); AUC: area under the curve.

Both the *first follow‐up* (AUC 0.70; 95% CI: 0.51–0.88) and *delta* (AUC 0.69; 95% CI: 0.54–0.84) CHA₂DS₂‐VASc scores showed higher AUCs for stroke prediction than *baseline* CHA₂DS₂‐VASc score (AUC 0.63; 95% CI: 0.44–0.82); however, these differences did not reach statistical significance by DeLong's test (F1 vs. baseline: *p* = 0.082; delta vs baseline: *p* = 0.621) (Figure 3A).

### 3.4 Comparing the Predictive Performance of Baseline, First Follow up, and Delta HAS‐BLED Scores

*Delta* HAS‐BLED score showed the highest discrimination (AUC 0.73, 95% CI 0.55–0.91), exceeding both *first follow‐up* (AUC 0.60, 95% CI 0.40–0.80) and *baseline* (AUC 0.58, 95% CI 0.43–0.74) (Figure 3B). However, neither comparison with baseline reached statistical significance by DeLong's test (*first follow‐up* vs *baseline*: *p* = 0.926; *delta* vs *baseline*: *p* = 0.176).

### 3.5 Multistate Model Analysis

In multivariable regression, those who recently transitioned to high risk between baseline and first follow‐up had a higher risk of stroke compared with those who were stably high since baseline (adjusted OR 5.83, 95% CI 0.47–46.1), although this did not reach statistical significance (Supporting Information S1: Table 1).

In multivariable logistic regression, compared to patients with stable HAS‐BLED score at baseline and follow‐up, an increase in HAS‐BLED score from baseline to first follow‐up was significantly associated with 1‐year bleeding risk (adjusted OR 15.20, 95% CI 2.01–119.00) (Supporting Information S1: Table 1).

## 4 Discussion

The principal findings of our study are as follows: (i) Bleeding and stroke risks are dynamic and change over time in patients with AF; (ii) *delta* and *First follow‐up* CHA₂DS₂‐VASc scores achieved stronger predictive performance compared to *baseline* CHA₂DS₂‐VASc score in predicting 1‐year stroke risk; (iii) *delta* HAS‐BLED score had the higher AUC compared to *baseline* HAS‐BLED score; (iv) Patients who recently transitioned to a high CHA₂DS₂‐VASc score had a higher 1‐year stroke risk than those who had remained high risk from baseline.

Our findings regarding that stroke and bleeding risks in patients with AF are dynamic but not statistic have been confirmed results of previous studies [11, 12, 13]. Analysis of the 1361 patients with AF at baseline, after 2 years, and after 4 years revealed that both the CHA₂DS₂‐VASc and HAS‐BLED scores are dynamic and change over time [12]. Another prospective longitudinal study enrolling 167,262 patients with AF, significant increase in proportion of patients at high risk of stroke was observed, after follow‐up of 3 years [13]. Moreover, it has been shown that HAS‐BLED score is also dynamic in nature and can increase over time in patients with AF [11]. Taken together, it must be recognised that bleeding and stroke risk such as CHA₂DS₂‐VASc and HAS‐BLED scores change over time in patients with AF.

Our study demonstrated that *delta* and *first follow‐up* CHA₂DS₂‐VASc scores had higher AUCs comparing to *baseline* CHA₂DS₂‐VASc score in predicting stroke risk. These findings are in good agreement with results of previous studies [12, 13]. It has been shown that 2‐year CHA₂DS₂‐VASc score had better predictive performance compared to baseline CHA₂DS₂‐VASc score in predicting ischemic stroke/TIA in patients with AF [12]. In good agreement with our findings, in cohort of 1127 AF patients, follow‐up and Delta CHA_2_DS_2_‐VASc scores were significantly associated with ischemic stroke [14].

Moreover, we demonstrated that Patients who transitioned to a high CHA₂DS₂‐VASc score from baseline to first follow‐up, exhibited a higher likelihood of stroke at 1 year than those who had been stably high risk since baseline, suggesting that recent risk increase may confer a period of greater susceptibility. In good agreement with our findings, the recent development of new risk factors for stroke was associated with a higher subsequent stroke risk compared with patients who had a stable CHA₂DS₂‐VASc score [15]. In addition, in another cohort of patients with AF, the slope of the change in the CHA₂DS₂‐VASc score was associated with an increased risk of ischemic stroke, with higher slopes corresponding to greater risk, further supporting the dynamic nature of stroke risk in AF patients [16].

Based on the aforementioned findings and our study, it is evident that the CHA₂DS₂‐VASc score in AF patients is dynamic, reinforcing the need for closer follow‐ups and regular risk reassessments to improve stroke prevention and patient management [17]. The 2024 ESC guidelines [18], emphasize the importance of evaluation and dynamic reassessment as part of the “E” component in the AF‐CARE framework, but they do not specify an optimal timeframe for reassessment. In contrast, the 2021 APHRS guidelines recommend stroke risk reassessment at least each year and every 3‐4 months if possible [19, 20, 21]. Further larger studies with longer follow‐up are warranted to evaluate optimal time periods for stroke risk reassessment in patients with AF.

In our study, *delta* HAS‐BLED score had the better predictive ability compared to *baseline* HAS‐BLED score. Analysis of 19,566 patients with AF after follow‐up of 93,783 person‐years demonstrated that Follow‐up HAS‐BLED score achieved higher AUCs compared to baseline HAS‐BLED score [11]. Therefore, ongoing and periodic bleeding risk assessment in patients with AF should be implemented, considering the better predictive performance of HAS‐BLED score at follow‐up and its dynamic nature. Bleeding events are also highly predictive of subsequent adverse clinical cardiovascular events [22], and bleeding risk per se is the interaction of modifiable and non‐modifiable bleeding risk factors [23], including the so‐called ‘East Asian’ paradox regarding bleeding and antithrombotic therapy. However, the latest guidelines [18, 19, 20, 24] indicate physicians to reassess the bleeding risk in patients with AF, but they do not specify any particular time intervals for such reassessment. Therefore, further studies are warranted to determine the optimal timing for bleeding risk reassessment in this population.

Given our findings, bleeding and stroke risks should be regards as dynamic in nature rather than static. Therefore, instead of relying solely on baseline assessments, physicians should regularly reassess these risks to support informed clinical decision making, as balancing stroke prevention and bleeding risk is a critical component of optimal care in patients with AF.

Our study has several limitations that should be acknowledged. First, its observational design may introduce biases and limit the generalizability of the findings to broader populations. The limited sample size and relatively short follow‐up, along with potential differences in anticoagulation patterns, selection bias, registry‐specific characteristics, residual confounding, and limited statistical power due to few events, likely contributed to the wide confidence intervals observed in our regression models. Although not statistically significant, the unexpectedly lower embolic risk observed in patients with persistent or permanent AF compared with those with paroxysmal AF contradicts established evidence and may be explained by these factors; therefore, this finding should be interpreted with caution. Indeed, ethnic differences in AF‐related complications such as stroke and bleeding have been reported [25, 26]. Furthermore, the short follow‐up period may not fully capture long‐term trends in risk progression, emphasizing the need for larger, multicenter studies with extended follow‐up durations to validate our findings.

## 5 Conclusion

Regular reassessment of CHA₂DS₂‐VASc and HAS‐BLED scores is crucial in AF patients, as both stroke and bleeding risks evolve over time. This highlights the importance of dynamic risk stratification to ensure timely and appropriate management. While current guidelines emphasize periodic evaluation, they do not provide specific recommendations on optimal reassessment intervals. Further research is needed to establish evidence‐based timeframes for re‐evaluating stroke and bleeding risk, which could enhance clinical decision‐making and improve patient outcomes.

## Author Contributions

**Amir Askarinejad:** formal analysis, methodology, writing – original draft, writing – review and editing, visualisation. **Tommaso Bucci, Enrico Tartaglia**, and **Michele Rossi:** writing – review and editing. **Gregory Y. H. Lip:** conceptualisation, methodology, project administration, and supervision. **Majid Haghjoo:** supervision.

## Conflicts of Interest

G.Y.H.L. is a NIHR Senior Investigator and coprincipal investigator of the AFFIRMO project on multimorbidity in AF, which has received funding from the European Union's Horizon 2020 research and innovation program under grant agreement No 899871. All other authors have reported that they have no relationships relevant to the contents of this paper to disclose.

## Supporting information


**Supplementary figure 1:** Study flowchart. **Supplementary Table 1:** Multivariable Logistic Regression of Stroke and Bleeding at 12 Months According to Changes in CHA₂DS₂‐VASc and HAS‐BLED Scores.

## Data Availability

The data that support the findings of this study are available from the corresponding author upon reasonable request.
