# Supplementary material for: Stroke and Bleeding Risks in Atrial Fibrillation Are Not Static but Dynamic: A Dynamic Assessment of CHA2DS2‐VASc and HAS‐BLED Scores in the Iranian Registry of Atrial Fibrillation (IRAF)
Source: J Cardiovasc Electrophysiol. 2025 Nov 20;37(1):110–8. doi: 10.1111/jce.70190 (PMC12794774; doi:10.1111/jce.70190)
Supplement: Supplementary file 1 — Supplementary figure 1: Study flowchart. Supplementary Table 1: Multivariable Logistic Regression of Stroke and Bleeding at 12 Months According to Changes in CHA₂DS₂‐VASc and HAS‐BLED Scores. [file JCE-37-110-s001.docx]

**Stroke and Bleeding Risks in Atrial Fibrillation are not static but dynamic:**

**A Dynamic Assessment of CHA_2_DS_2_-VASc and HAS-BLED Scores in the Iranian Registry of Atrial Fibrillation (IRAF)**

Amir Askarinejad^1^, Tommaso Bucci^1,2^, Enrico Tartaglia^1^, Michele Rossi^1^, Gregory Y. H. Lip^1,3,4^*****, Majid Haghjoo^5,6^*****

Supplementary Materials


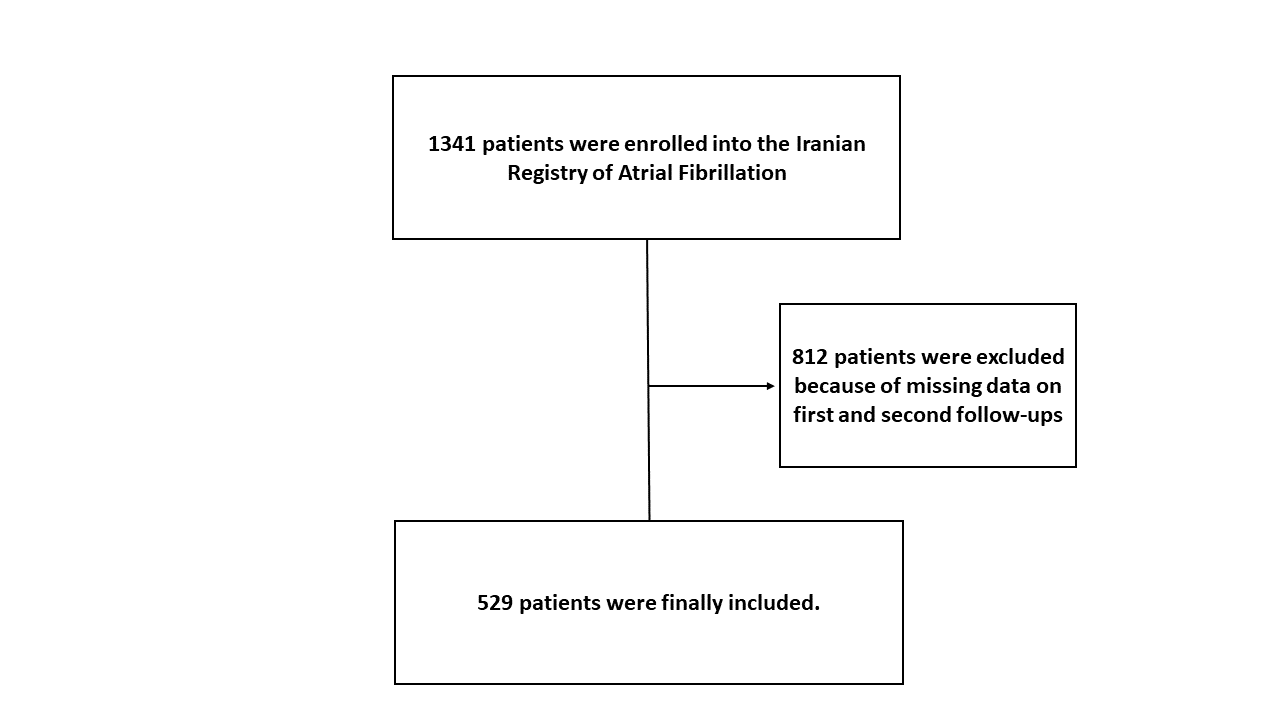


Supplementary figure 1. Study flowchart.

Supplementary Table 1. Multivariable Logistic Regression of Stroke and Bleeding at 12 Months According to Changes in CHA₂DS₂-VASc and HAS-BLED Scores

| **Variable** | | | **OR (95% CI)** |
| --- | --- | --- | --- |
| **1-year stroke risk prediction** | | | |
| **CHA₂DS₂-VASc risk-trajectory groups** | **Stable High Stroke Risk (0–6 months)** | | Ref |
|  | **Newly High Stroke Risk (0–6 months)** | | 5.83 (0.47 – 46.1) |
| **AF Type** | **Paroxysmal** | | Ref |
|  | **Permanent** | | 0.30 (0.01 – 5.27) |
|  | **Persistent** | | 0.60 (0.10 – 2.55) |
| **CKD** | | | 1.79 (0.18 – 9.33) |
| **OAC** | | | 0.20 (0.04 – 0.78) |
| **LVEF (per 1% increase)** | | | 0.97 (0.93 – 1.02) |
| **1-year bleeding risk prediction** | | | |
| **HAS-BLED risk-trajectory groups** | **Stable HAS-BLAED Score (0–6 months)** | | Ref |
|  | **Upward transition in HAS-BLED Score category (0–6 months)** | | 15.20 (2.01 – 119.00) |
|  | **Downward in HAS-BLED Score category (0–6 months)** | | 5.86 (0.03 – 147.00) |
| **AF Type** | **Paroxysmal** | | Ref |
|  | **Permanent** | | 3.68 (0.03 – 76.50) |
|  | **Persistent** | | 9.94 (1.82 – 104.0) |
| **OAC** | | | 1.32 (0.25 – 8.41) |
| **LVEF (per 1% increase)** | | | 1.05 (0.98 – 1.17) |
| **Age** | | | 1.02 (0.96 – 1.09) |
| **Gender** | | **Male** | Ref |
|  |  | **Female** | 1.09 (0.21 – 5.60) |

AF: Atrial fibrillation; CI: Confidence interval; CKD: Chronic kidney disease; OAC: Oral anticoagulant; OR: Odds ratio; LVEF: Left ventricular ejection fraction.
